# Evaluation of CD44 and TGF-B Expression in Oral Carcinogenesis

**DOI:** 10.30476/DENTJODS.2020.84393.1079

**Published:** 2021-03

**Authors:** Narges Ghazi, Nasrollah Saghravanian, Mohammad Taghi Shakeri, Mounes Jamali

**Affiliations:** 1 Dept. of Oral and Maxillofacial Pathology, School of Dentistry, Mashhad University of Medical Sciences, Mashhad, Iran; 2 Dept. of Community Medicine and Public Health, Mashhad University of Medical Sciences, Mashhad, Iran; 3 Postgraduate Student of Periodontology, School of Dentistry, Mashhad University of Medical Sciences, Mashhad, Iran

**Keywords:** Oral squamous cell carcinoma, Dysplasia, CD44, TGF-B

## Abstract

**Statement of the Problem:**

Oral squamous cell carcinoma (OSCC) is the most common malignancy of the oral cavity. Early diagnosis of OSCC by using biomarkers provides preventive treatment approach to suppress the disease in early stages. CD44 as a cancer stem cell (CSC) marker may be cleaved by MT1-MMP and plays an important role in migration of cancer cells. TGF-B promotes formation of invasive cancer cells phenotype through epithelial mesenchymal transition (EMT) and induces MT1-MMP formation.

**Purpose:**

The aim of this study was to evaluate the expression of TGF-B and CD44 in leukoplakia (premalignant lesion), squamous cell carcinoma (SCC), and normal oral mucosa to determine the role of these markers in the carcinogenesis process of the oral mucosa.

**Materials and Method:**

In this retrospective study, the expression of TGF-B and CD44 were evaluated in 55 paraffin-embedded specimens (10normal mucosa, 15 non-dysplastic leukoplakia, 15 dysplastic leukoplakia, and 15 OSCC) by immunohistochemistry. Statistical analyses were performed using Kruskal-Wallis, Mann-Whitney, and Spearman’s rank correlation tests.

**Results:**

Evaluation of CD44 and TGF-B expression in the four studied groups showed statistical significant difference for each marker (*p*< 0.001). Pairwise comparison of CD44 and TGF-B expression in all groups except normal mucosa and non-dysplastic leukoplakia demonstrated statistical significant difference. In addition, there was positive significant correlation between two markers (r= 0.914, *p*< 0.001). Diagnostic test’s accuracy for identification of OSCC and dysplastic leukoplakia from non-dysplastic leukoplakia and normal tissues and recognition of OSCC from dysplastic leukoplakia showed optimum sensitivity and specificity.

**Conclusion:**

Increased expression of CD44 as a cancer stem cell marker and TGF-B as an EMT marker from normal mucosa to non-dysplastic leukoplakia, dysplastic leukoplakia, and OSCC and also the significant correlation between these two markers indicated their role in carcinogenesis of oral mucosa.

## Introduction

Squamous cell carcinoma (SCC) is the most common malignancy of the oral cavity. It accounts for more than 90% of all oral malignancies [ 1 ]. It has a morbidity and mortality rate of around 50% [ 2 ]. Despite the advances in novel therapeutic modalities, the mortality and morbidity rate of oral squamous cell carcinoma (OSCC) has not been significantly decreased in the recent years [ 3 ]. Epithelial carcinogenesis is a multi-phase process [ 4 ]. It is believed that initial changes occur in normal oral epithelium, which causes progression to oral dysplasia and cancer due to several genetic mutations. Oral carcinoma is the final outcome of this multi-step process that often occurs prior to morphological changes of the epithelium. Therefore, morphological changes do not always predict the possible progression of dysplasia to cancer. Several multi-functional factors may play a role in progression and transformation of potentially malignant oral lesions to OSCC [ 5 ].

In recent years, various markers related to cancer stem cells (CSCs) have been identified, which affect the initiation, progression, and treatment resistance of cancers. CD44 is a cell surface glycoprotein and, as a CSC marker, plays an important role in cell migration and adhesion, tumor invasion, prognosis, and metastasis of cancers [ 2 , 6 ].

Epithelial mesenchymal transition (EMT) is an important factor for the initiation of invasive cancer cell phenotype and transition to carcinoma. Transforming growth factor-beta (TGF-B) is among the most important cytokines that induce EMT. TGF-B belongs to superfamily consist of more than thirty proteins including growth factors, bone morphogenetic proteins, and activins. TGF-B as a multi-functional cytokine plays an important role in proliferation, differentiation, and migration of cancer cells as well as cancer progression and metastasis of cancers [ 7 - 9 ].

Recently, it has been shown that CD44 is expressed in cancer cells and may be cleaved at the ectodomain by membrane type 1- matrix metalloproteinase (MT1-MMP), forming soluble CD44. Thus, it plays a critical role in cancer cell migration. TGF-B is among the markers that can induce MT1-MMP [ 10 ]. Therefore, further investigations on molecular diagnostic biomarkers including EMT and CSC markers may provide useful information about detection of malignant transformation.

To the best of our knowledge, this study is the first to assess simultaneous expression of these two markers in leukoplakia as a potentially malignant oral lesion. The aim of this study was to investigate the expression of TGF-B as an EMT inducing marker and CD44 as a CSC marker in the process of carcinogenesis in oral mucosa.

## Materials and Method

### Patients

This retrospective study evaluated 55 specimens inclu-ding 15 specimens of OSCC (11 males and 4 females), 15 specimens of dysplastic leukoplakia (8 males and 7 females), 15 specimens of non-dysplastic leukoplakia (4 males and 11 females) and 10 specimens of normal mucosa (normal mucosa of non-inflammatory hyperplastic epithelial or connective tissue lesions in 5 males and 5 females). The specimens were retrieved from the archives of the Oral and Maxillofacial Pathology Department, Mashhad University of Medical Sciences. The Ethical Committee of Mashhad University of Medical Sciences provided ethical approval for this study (IR.MUMS. DENTISTRY.REC.930943.). Demographic information such as age and sex of patients were also retrieved from the patient files.

The inclusion criteria were based on final pathology report of OSCC and histopathological diagnosis of dysplastic and non-dysplastic oral epithelium with clinical diagnosis of leukoplakia. The diagnosis was confirmed by two pathologists. Inadequately or improperly fixed tissues were excluded.

### Immunohistochemical staining for CD44 and TGF-B 

Four-µm sections of formalin-fixed paraffin-embedded tissue were submitted for immunohistochemical evaluation. Tissue sections were de-paraffinized in xylene and rehydrated using descending concentrations of ethanol. Next, the sections were boiled in 0.01 citrate buffer with a pH of 6.0 for 10 minutes for antigen retrieval. Methanol along with 0.5% hydrogen peroxide was used for 10 minutes to block the activity of endogenous peroxidase. The tissue sections were rinsed with Tris-buffered saline with a pH of 7.6 and incubated in diluted normal serum for 10 minutes. They were then treated with primary antibody for 30 minutes according to the manufacturer’s instructions. The anti-CD44 (Novocastra NCL-CD44-2, clone DF1485, 1:50 dilution, Novocastra Laboratories, Newcastle, UK) and anti-TGF-B (NCL-TGF B, Novocastra laboratories, Newcastle upon tyne/UK, 1:50 dilution) monoclonal antibodies were then applied. The sections were rinsed with Tris-buffered saline and incubated with the secondary antibody. They were then washed with Tris-buffered saline and reacted with di-amino benzene hydrochloride for 5 minutes. Finally, the slides were counterstained with hematoxylin and cover-slipped using a synthetic mounting medium. Sections of tonsillar tissue for CD44 and human placenta for TGF-B were used as positive control. Omission of the primary antibody served as the negative control.

Two pathologists evaluated immunohistochemically-stained slides under a light microscope (400×). Membranous staining and cytoplasmic staining were regarded positive for CD44 and TGF-B respectively. The immunoreactivity of the cells was evaluated under light microscope (Labomed) at a final magnification of 400×.

According to the percentage of positive cells, immunoreactivity (designated as labeling index LI) was scored as follows: Immunoreactivity for CD44 [ 11 ]:

4+, 75-100%; 3+, 50-75%; 2+, 25-50%; 1+, 5-25% and negative (-), less than 5%of epithelial cells were positively stained in cell membrane. Immunoreactivity for TGF-B [ 12 ]: 

3+, 50-100%; 2+, 20-50%; 1+, less than 20%; negative (-), none of epithelial cells were positively stained in the cytoplasm.

### Statistical analysis

Data were analyzed using SPSS version 16. The Kruskal-Wallis test was used to compare the expression of TGF-B and CD44 markers among the four groups. The Mann-Whitney test was used for pairwise comparisons of the groups. The Spearman’s correlation coefficient was applied to assess the correlation between the two markers in each studied group. The receiver operating characteristic (ROC) curve was drawn to determine the cutoff point. 

## Results

**Figure 1:**
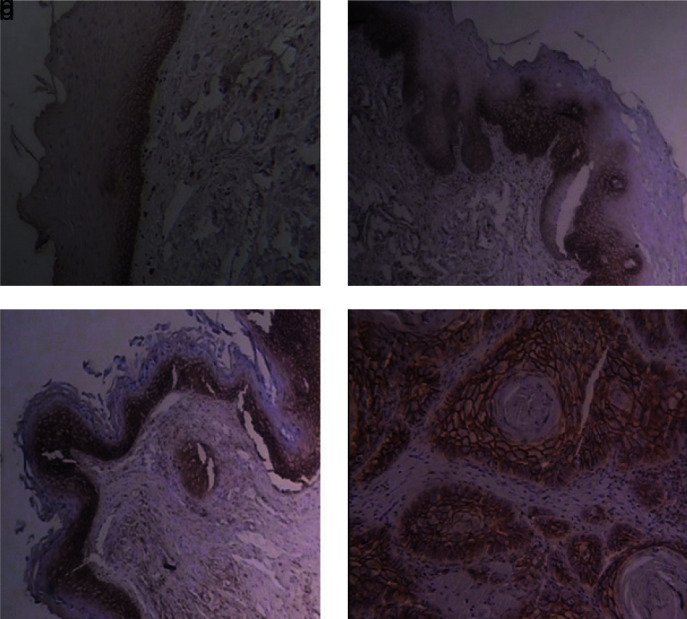
Membranous immunoreactivity for CD44 in **a:** normal oral mucosa (1+), x100, **b:** non-dysplastic leukoplakia(1+), x100, **c:** dysplastic leukoplakia(2+), x100, d: OSCC(3+),x400

This study evaluated 15 OSCC, 15 non-dysplastic oral leukoplakia, 15 dysplastic oral leukoplakia and 10 normal mucosa samples. The patients included 28 males and 27 females between 15 to 90 years. The mean ages of patients were 55.33, 55.29, 56.13 and 42.10 years in OSCC, non-dysplastic oral leukoplakia, dysplastic oral leukoplakia and normal mucosa groups respectively. Significant difference was not observed for age (*p*= 0.256) and gender (*p*= 0.194) among studied groups. Comparison of CD44 expression in the studied groups showed that the greatest mean total score was in OSCC group (3.87) and the lowest was in normal mucosa group (0.7). In addition, non-dysplastic and dysplastic leukoplakia lesions showed mean total score of 1.0 and 2.20, respectively (Table 1, Figure 1). 

**Table 1:** Comparison of the mean total score of CD44 expression in the studied groups

Lesion	Mean total	score	Std. deviation	Number	CD 44 LI				
					0	1+	2+	3+	4+
Normal	0.70	0.48	10	3(30.0%)	7(70.0%)	0	0	0
Non-dysplastic oral leukoplakia	1.00	0.53	15	2(13.3%)	11(73.3%)	2(13.3%)	0	0
Dysplastic oral leukoplakia	2.20	0.41	15	0	0	12(80.0%)	3(20.0%)	0
OSCC	3.87	0.35	15	0	0	0	2(13.3%)	13(86.7%)
*p* Value				*p*< 0.001				

**Figure 2:**
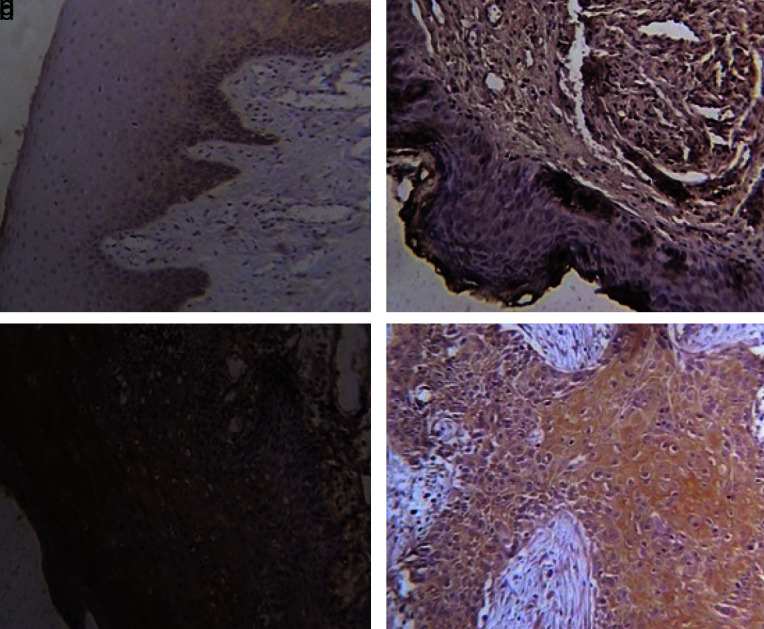
Cytoplasmic immunoreactivity for TGF-B in (A): normal oral mucosa (1+), x100, (B): non-dysplastic leukoplakia (1+) x100, (C): dysplastic leukoplakia (2+), x400, (D): OSCC (3+), x400

According to the Kruskal-Wallis test, a significant difference was observed among the groups (*p*< 0.001). Pairwise comparison of CD44 expression in the studied groups was performed. All differences were found to be significant (*p*< 0.001) except comparison of non-dysplastic leukoplakia and normal mucosa (*p*= 0.285). Comparison of TGF-B expression among the four groups was performed according to the mean total score. OSCC lesions showed greatest mean total score (2.80), while normal mucosa demonstrated lowest mean total score (0.80). In addition, non-dysplastic and dysplastic leukoplakia lesions showed a mean total score of 1.33 and 2.19, respectively (Table 2, Figure 2). According to the Kruskal-Wallis test, a significant difference was seen among the groups (*p*< 0.001). The Spearman’s non-parametric test was used to assess the correlation between the expression of CD44 and TGF-B, which indicated a positive significant association between the expression of CD44 and TGF-B (*p*< 0.001, r= 0.914).

**Table 2:** Comparison of the mean total score of TGF-B expression in the studied groups

Lesion	Mean total score	Std. deviation	Number	TGF-β LI			
				0	1+	2+	3+
Normal	0.80	0.42	10	2(20.0%)	8(80.0%)	0	0
Non-dysplastic oral leukoplakia	1.33	0.49	15	0	10(66.7%)	10(66.7%)	0
Dysplastic oral leukoplakia	2.19	0.54	15	0	1(6.7%)	11(73.7%)	3(20.0%)
OSCC	2.80	0.41	15	0	0	3(20.0%)	12(80.0%)
*p* Value	*p*< 0.001						

Diagnostic accuracy of CD44 and TGF-B for differentiation of SCC and dysplastic leukoplakia from non-dysplastic leukoplakia and normal mucosa was assessed. The ROC curve was used after combining the “normal and non-dysplastic leukoplakia groups” and also “dysplastic leukoplakia and SCC” groups. The results showed that the accuracy of CD44 and TGF-B diagnostic markers for differentiation of SCC and dysplastic leukoplakia” from “non-dysplastic leukoplakia and normal mucosa” was 0.984 (95% CI: 0.957- 1.011, *p*< 0.001) and 0.935 (95% CI: 0.871-0.998, *p*< 0.001), respectively, and the cutoff point of 1.5 for both tests demonstrated maximum sensitivity and specificity.

Diagnostic accuracy of CD44 and TGF-B for differentiation of SCC from dysplastic leukoplakia was assessed. The ROC curve was specifically used for SCC and dysplastic leukoplakia. The results showed that the accuracy of CD44 and TGF-B diagnostic markers for differentiation of SCC from dysplastic leukoplakia was 0.984 (95% CI: 0.958-1.016, *p*< 0.001) and 0.807 (95% CI: 0.643-0.971, *p*< 0.01), respectively. The cutoff point of 3.5 and 2.5 showed maximum sensitivity and specificity for the two tests.

## Discussion

OSCC is the most prevalent oral cancer [ 1 ]. Diagnosis of OSCC in the preliminary phase by using biomarkers provides preventive treatment approach to suppress the disease in early stages. Thus, this study aimed to assess the expression of CD44 and TGF-B in OSCC and leukoplakia as a premalignant oral lesion.

Recent evidence has shown that only small subgroups of cells, referred as CSCs, with self-renewal capacity are responsible for tumor initiation and progression [ 13 - 14 ]. Thus, tumors are maintained by their own stem cells also known as self-sustaining cells [ 15 - 16 ]. 

 A number of studies [ 3 , 6 , 17 ] reported that head and neck SCC comprises a heterogeneous cell population with CSC-like properties such as high tumor seeding ability and resistance to chemotherapy and radiotherapy. Similar to other cancer cells, CSCs of head and neck SCC express surface markers such as CD44 and CD133, which are used for identification [ 3 , 6 , 17 ]. Several molecular studies considered CD44 as a candidate stem cell marker in normal squamous epithelium and SCC [ 6 , 13 , 17 ]. Chen *et al*. [ 6 ] suggested CD44 could promote drug resistance through anti apoptotic mechanisms. The functional role of CD44 is pleiotropic, includes inducing EMT, altering the cellular cytoskeleton, by promoting drug resistance, and through anti-apoptosis mechanisms.This protein can interact with numerous different ligands such as hyaluronate, fibronectin, and collagen. Thus, it has an important role in cell-cell and cell-matrix interaction as well as cell migration and tumor progression. It should be noted that EMT process has been the focus of various studies [ 6 , 9 , 14 , 18 ]. During the process of EMT, epithelial cells down-regulate the expression of cell-adhesion proteins and gain migratory and invasive properties. EMT is essential for numerous physiological processes such as fetus development, wound healing, fibrosis, progression of carcinoma, and metastasis [ 14 , 18 - 19 ]. A correlation has been found between the EMT and invasive behavior of different types of cancers including colon cancer, head and neck squamous cell carcinoma and so on [ 6 - 7 , 18 ]. It has been reported that EMT plays a fundamental role in development of a malignant phenotype [ 19 ].

TGF-B is one of the most important cytokines that induces EMT. The EMT process is a topic of interest in cancer research due to its correlation with progression of different tumor types. In the recent years, the relationship of EMT with CSCs is an important aspect of various studies [ 14 , 17 , 19 ]. 

This study aimed to assess the expression of TGF-B as an EMT-inducing marker and CD44 as a CSC marker in the process of carcinogenesis in oral mucosa and the correlation between these two markers. 

In the present study, the expression of CD44 was found to be significantly different among the four groups. Pairwise comparison of the groups for CD44 expression revealed significant differences in all cases except between patients with non-dysplastic leukoplakia and normal oral mucosa. A Significant increase in percentage of staining of CD44 non-dysplastic leukoplakia compared to dysplastic leukoplakia and OSCC indicated the connotation of these markers in the process of malignant transformation and detection of these lesions. The current results were in line with those of other studies on cancer of other organs and increased expression of CD44 from normal mucosa to dysplasia and cancer has been previously reported in esophageal and gastric cancers [ 20 - 21 ]. 

It should be noted that according to the current results, CD44 had maximum sensitivity and specificity for differentiation of “SCC and dysplastic leukoplakia” from “non-dysplastic leukoplakia and normal mucosa” as well as differentiation of SCC from dysplastic leukoplakia. This finding indicated the correlation between the expression of this cell adhesion molecule as a CSC antigen and transition to OSCC. 

Similar to the present study, the results of Abdulmajeed *et al*. [ 22 ], Rautava *et al*. [ 23 ], Oliveira *et al*. [ 2 ], and Bahar *et al*. [ 24 ], confirmed that increased expression of CD44 would increase the risk of malignancy. 

On the other hand, González-Moles *et al*. [ 25 ], Chang *et al*. [ 26 ], Gao *et al*. [ 27 ] and Godge *et al*. [ 4 ] showed down-regulation of this marker in malignant lesions. 

Moreover, assessment of the level of expression of TGF-B revealed a significant difference among the four study groups. Pairwise comparisons of the groups in this respect revealed significant differences in all comparisons except for the comparison between non-dysplastic leukoplakia and normal mucosa. It should be noted that according to the current results, TGF-B had maximum sensitivity and specificity for differentiation of “SCC and dysplastic leukoplakia” from “non-dysplastic leukoplakia and normal mucosa” as well as for differentiation of SCC from dysplastic leukoplakia. This finding indicated the role of expression of this marker as an EMT marker in malignant transformation of normal mucosa to OSCC.

Chen *et al*. [ 28 ] assessed the expression of TGF-B and MMPs in normal mucosa, non-atrophic lichen planus, atrophic lichen planus, and OSCC. The results showed upregulation of MMP9 and MMP11 by transition from normal mucosa to OSCC. Increased expression of TGF-B was also noted, which highlighted the role of this marker in regulation of MMPs.

Wagner *et al*. [ 29 ], and Chang *et al*. [ 26 ], showed that TGF-B expression would significantly increase from normal mucosa to leukoplakia and from leukoplakia to OSCC. Iamaroon *et al*. [ 8 ] assessed the expression of SAMD4, which is a TGF-B signaling molecule, in normal mucosa and OSCC using immunohistochemistry and the western blot. They observed that lack of expression of SMAD4 caused irregularities in TGF-B pathway and increased the carcinogenic properties of OSCC. To the best of our knowledge, this study was the first to assess the simultaneous expression of CD44 and TGF-B in premalignant oral lesions and OSCC. The results showed upregulation of both markers from normal mucosa to dysplastic epithelium and OSCC and found a significant correlation between these two markers. Further studies seem to be necessary on the function and expression of CD44 and TGF-B as therapeutic targets for premalignant oral lesions and OSCC. 

Mima *et al*. [ 30 ], Ouhtit *et al*. [ 31 ], Park *et al*. [ 32 ], Anido *et al*. [ 33 ] and Bourguignon *et al*. [ 34 ] highlighted the important role of CD44 in induction of mesenchymal phenotype mediated by TGF-B such that inhibition of CD44 would inhibit this phenotype and prevent the invasive nature of carcinoma. 

Further studies are required to investigate the function and expression of CD44 and TGF-B as therapeutic targets for management of premalignant oral lesions and OSCC.

## Conclusion

Increased expression of CD44 as a CSC marker and TGF-B as a marker involved in EMT from normal mucosa to non-dysplastic leukoplakia, dysplastic leukoplakia and OSCC and the significant correlation between these two markers indicated their role in carcinogenesis of oral mucosa. The markers evaluated in this study showed maximum sensitivity, specificity, and diagnostic accuracy for differentiation of “OSCC and dysplastic leukoplakia” from “non-dysplastic leukoplakia and normal mucosa” and also for differentiation of OSCC from dysplastic leukoplakia. 
